# High Selection Pressure Promotes Increase in Cumulative Adaptive Culture

**DOI:** 10.1371/journal.pone.0086406

**Published:** 2014-01-29

**Authors:** Carolin Vegvari, Robert A. Foley

**Affiliations:** Leverhulme Centre for Human Evolutionary Studies, Department of Archaeology and Anthropology, University of Cambridge, Cambridge, United Kingdom; University of Warwick, United Kingdom

## Abstract

The evolution of cumulative adaptive culture has received widespread interest in recent years, especially the factors promoting its occurrence. Current evolutionary models suggest that an increase in population size may lead to an increase in cultural complexity via a higher rate of cultural transmission and innovation. However, relatively little attention has been paid to the role of natural selection in the evolution of cultural complexity. Here we use an agent-based simulation model to demonstrate that high selection pressure in the form of resource pressure promotes the accumulation of adaptive culture in spite of small population sizes and high innovation costs. We argue that the interaction of demography and selection is important, and that neither can be considered in isolation. We predict that an increase in cultural complexity is most likely to occur under conditions of population pressure relative to resource availability. Our model may help to explain why culture change can occur without major environmental change. We suggest that understanding the interaction between shifting selective pressures and demography is essential for explaining the evolution of cultural complexity.

## Introduction

Our capacity for cumulative adaptive culture is an essential aspect of modern human behavioural adaptations. The essence of cumulative culture is that elements are added, and this leads to greater complexity. Cultural complexity has been variously defined [1], [2], [3], [4], and can comprise both local changes in the scale of cultural items, and the general trends over time leading to living humans [5], [6]. In this paper we use the number of added traits as a proxy for cultural complexity; this might match best to technological and material complexity, but is not necessarily confined to it. In the archaeological record, we observe that technological complexity has not increased continuously, and extant human societies differ in the complexity of their technological systems. For example, early occurrences of more complex material cultural adaptations in South Africa during the Middle Stone Age seem to have been short-lived and followed by societies with comparatively less complex material culture [7]. In the ethnographic record, societies range in their technological complexity, from the basic tool kit of the Tasmanians to state societies with a tool kit that in its entirety can no longer be produced or handled by a single individual [2], [8].

Given the general human capacity for cumulative culture [9], [10], the obvious question is: Why are some human cultures more complex than others? Factors that have been proposed to affect the level of cultural complexity of a population are risk [11], population size and connectivity [12], [13], [14], [15], [16], and the relative costs and benefits of innovating and maintaining cultural traits [17], [18], [19]. Recent studies have emphasised the role of population size and social connectivity in promoting adaptive culture [12], [13], [14], [15], [20]. However, the empirical evidence for this relationship is contentious [11], [21].

The role of selection in the evolution of cumulative adaptive culture has been assumed but not well-explored, even though selection, by definition, should be a major driving force in the evolution of adaptive traits [22], [23]. Our aim in this paper is to investigate the relative importance of natural selection and demography and their interaction in the evolution of cumulative adaptive culture. For this purpose, we use a spatially explicit agent-based simulation model that focuses on subsistence-related cultural traits.

We use this kind of model because it allows us to explicitly model human-environment interactions and human adaptations at the behavioural level. We focus on subsistence-related traits because their adaptive value is evident and the fitness benefits that they confer are relatively easy to assess. As a measure for cumulative adaptive culture we use the number of cultural traits per individual. This means we measure complexity as the size of a trait list. A cultural trait may correspond to a techno-unit, a tool, a specified piece of knowledge or behaviour, or the ability to achieve a goal in a given domain.

We expect that stronger selection pressure leads to an accelerated increase in the number of adaptive cultural traits. Selection is strong if the fitness gains associated with higher cultural complexity are high, this means the reproductive success of individuals with more traits is higher. The effect of selection can be overwhelmed by random drift. Selection is more likely to influence outcomes in larger populations, drift in smaller populations. We predict that even small or isolated populations should accumulate adaptive culture fast if selection is strong enough. Large and well-connected populations under high selection pressure should display the highest rates of increase in cumulative adaptive culture.

Contrarily, if the innovation of new cultural traits is expensive the accumulation of adaptive traits should proceed more slowly or be prevented. Innovation may be costly because individuals have to spend time and energy innovating that they could otherwise spend on different activities [19], [24]. The evolution of cumulative adaptive culture should also be slowed down by learning costs because cultural traits may be conceptually difficult and learning involves a lot of effort [25].

We distinguish between two types of innovation; the first are innovations of independent cultural traits that enable individuals to exploit a new resource; the second are innovations which represent modifications of existing traits and make individuals more efficient at exploiting a resource which has already been part of their subsistence spectrum before (see [26] for a similar distinction between different types of innovation).

The possibility of both types of innovation occurring within the same population may give rise to population fluctuations [27], [28]. The reason is that independent innovations increase the equilibrium population size by shifting it towards the absolute carrying capacity of a habitat (determined by the sum of all potentially exploitable resources [29]). At the same time improvements in the ability of individuals to extract resources may not necessarily lead to population growth and even lead to population decline. Thus, the dynamics of cultural transmission and innovation by themselves may be major determinants of demographic transitions in the absence of environmental change. This may help to explain why cultural transitions in the archaeological record may or may not coincide closely with changes in environmental conditions [7], [30], [31]. We tested the above hypotheses with our model. The results indicate that natural selection is a non-negligible force in the evolution of cultural complexity.

## Methods

*(For a full model description according to the ODD standard protocol for agent-based models* [32], *see Text S1. See Figure S4. See Table S17 for an overview of the model parameters and the values used in the simulations. Please email the corresponding author for source code and data files.)*

We propose an agent-based simulation model in which the behaviour and combined characteristics of low-level entities, agents as representations of human individuals, give rise to observable properties on higher organisational levels. Each individual is a member of a group, and all groups together constitute the total population. For simplicity groups are not internally structured in this set of analyses, but since individuals are born into their parents' group and form part of a family unit, the concept of groups in the model is close to that of hunter-gatherer bands or units composed of several bands [33].

As we are interested in modelling human-environment interactions that can lead to subsistence-related selection pressure, we consider our model population within a simple environmental framework (a two-dimensional grid of 10×10 squares). We use a fixed area for a hunter-gatherer group until it reaches carrying capacity, so population size translates into population density in the simulation. Individuals can invent or learn cultural traits that enable them to consume specific environmental resources. There are ten environmental resources, and individuals can acquire up to ten traits. Individuals only learn or innovate if they sense that they are under resource pressure, this means if they cannot meet their resource requirements.

Individuals that have reached a minimum age of 15 years can reproduce sexually, if they find a partner and if they have a minimum energy score to pass on enough energy to the new-born individual. This means that the reproductive rate is linked to resource availability (see Text S1 for details.) Offspring initially learn their cultural traits from their parents (see SI for details). The error rate during copying a trait is 5%; this means that with a probability of 5% the learner copies a trait value that is either one unit higher or one unit lower than the trait value of the cultural model. Higher trait values correspond to higher skill levels (see below). In addition, individuals can learn from other group members, and members of neighbouring groups, depending on model parameters. To be conservative, we specify that an individual can only invent a new trait, if he/she has reached the maximum skill level at the trait that precedes the new trait in the trait list (this corresponds to a notion of experience). The invention of a new trait comes at a cost that is subtracted from the individual's energy score.

The amount of a resource that an individual can extract from the environment depends on the individual's skill with the corresponding cultural trait, and is calculated as follows:
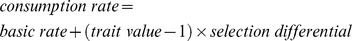
The basic rate is the resource-specific consumption rate if the cultural trait variant has a value of one. The selection differential is a key model parameter that determines the level of resource pressure and thereby the intensity of selection. It is defined as the increase in the resource extraction rate per one unit increase in the skill level at a particular cultural trait. Individuals can only consume resources for which they have a trait and cannot accumulate more than 50 resource units. They attempt to consume at least as many resource units as necessary to meet their minimum energy requirement at each time step (five resource units). Groups under resource pressure can migrate to another location and/or fission (see Text S1 for details).

Using the model outlined above we examined the effects of resource availability, social interactions between neighboring groups, selection intensity, and innovation and learning costs on the number of cultural traits per individual. Higher resource availability increases the population size. Individuals could either only interact with other individuals from within their own group (only learn from or find partners in their own square) or in addition with individuals from neighbouring squares (Moore neighborhood). Selection intensity was determined by the selection differential.

We used Bonferroni-corrected Wilcoxon rank sum tests on the end values of ten independent simulation runs to compare the average number of cultural traits per individual, mean group size and the level of competition among individuals in different populations. Competition was measured as the percentage of individuals in the total population that could not meet their minimum resource requirements.

## Results

**Figure 1:**
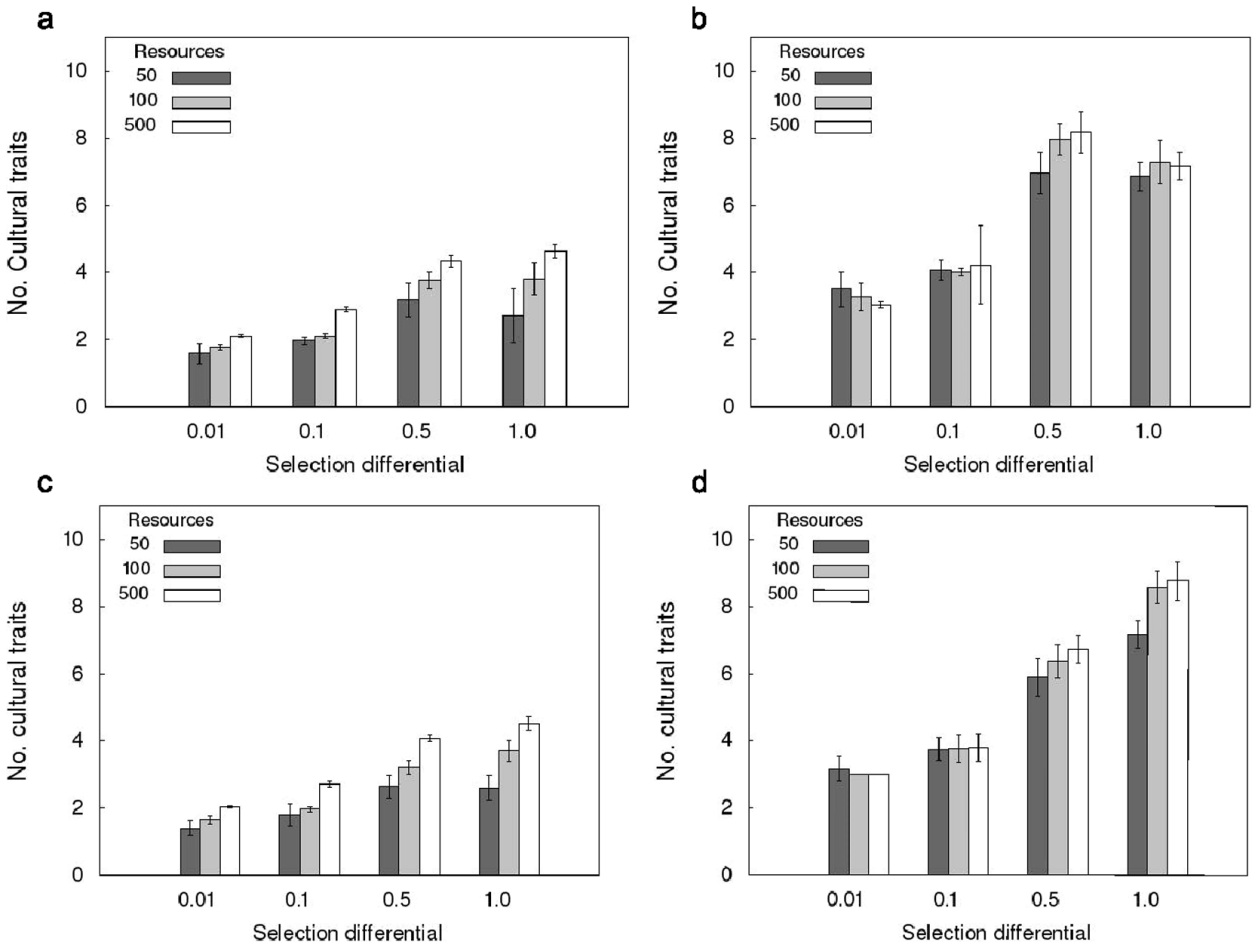
Effect of selection pressure, group size, interaction between groups and learning costs on the mean number of cultural traits per individual. a, b: no learning costs; c, d: learning costs 1 energy unit per learning event; a, c: isolated groups; b, d: interaction between neighboring groups. Legend indicates number of resource units per square. Results are grouped according to selection differential (x-axis). a, b: Higher selection pressure can increase the number of traits per individual up to a certain point (selection differential 0.5), but can lead to a decrease if it is too high (1.0). Higher resource availability significantly increases trait number for isolated groups and for interacting groups with intermediate selection differentials. Interacting groups always have a higher trait number than isolated groups. c, d: Learning costs can significantly decrease the number of traits per individual. If there are learning costs higher selection pressure always increases trait number (see text for details).

In populations with isolated groups increased selection pressure had a linear effect on cultural complexity. If the amount of resources stayed the same, higher selection pressure led to a higher number of cultural traits per individual compared to lower selection pressure (Fig. 1a). In isolated groups higher selection differentials were also associated with higher competition levels (Figure S1, Table S14). In contrast, if groups were not isolated, but individuals could interact with individuals from neighboring groups, selection pressure had a non-linear effect on cultural complexity. Stronger selection pressure only increased the number of traits per individual up to a point (selection differential 0.5). Beyond this point (selection differential 1.0) the number of cultural traits did not increase further and even declined (Fig. 1b; Table S1).

The effect of higher resource availability and concomitant larger group sizes (Figure S2, Table S5, Table S6) on the number of cultural traits was always significant for populations with isolated groups except for selection differential 0.01, for which small differences were not significant (Table S2). This may be explained by the increased importance of drift in small populations.

For populations with interacting groups, resource availability only significantly affected group sizes and trait numbers at selection differentials 0.1 and 0.5. Population sizes at very low (0.01) or very high (1.0) selection differentials were similar to each other, and lower than for intermediate selection differentials (0.1, 0.5) (Table 1). Connected groups always evolved significantly more cultural traits per individual and displayed higher competition levels than isolated groups (Table S3, Table S15, Table S16).

**Table 1:** Mean number of cultural traits ± standard deviation and mean group sizes in populations with isolated or interacting groups, with different selection differentials and resource availabilities.

	Isolated groups		Interacting groups	
Resource values	Group size	No. traits	Group size	No. traits
Selection differential 0.01				
50	12.4±2.530	1.58±0.297	39.1±5.940	3.50±0.514
100	30.6±2.293	1.78±0.088	71.5±8.450	3.27±0.413
500	197.3±3.108	2.09±0.044	333.2±4.929	3.04±0.103
Selection differential 0.1				
50	17.7±1.770	1.97±0.102	45.1±3.728	4.07±0.303
100	38.8±2.198	2.01±0.075	88.4±3.325	4.01±0.107
500	267.8±11.805	2.90±0.072	489.3±48.303	4.61±0.455
Selection differential 0.5				
50	24.5±3.396	3.19±0.492	62.6±12.204	6.95±0.626
100	56.3±4.132	3.77±0.247	168.5±9.581	7.95±0.478
500	291.6±9.523	4.34±0.167	789.5±92.558	8.16±0.620
Selection differential 1.0				
50	17.2±4.774	2.72±0.798	41.0±3.810	6.86±0.440
100	41.4±4.871	3.79±0.470	75.3±8.378	7.29±0.656
500	224.0±10.626	4.62±0.209	347.1±28.510	7.16±0.424

Maximum energy score of individuals was capped at 50. Cost of inventing a new trait was 10.

The effect of higher selection differentials on cultural trait number is partly due to higher mean group sizes if individuals consume more resources. However, in some cases populations with no significant differences in size evolved more cultural traits if they were exposed to stronger selection pressure. For example, in populations with interacting groups mean group sizes do not differ significantly for selection differentials 0.01 and 1.0, but the numbers of traits per individual is significantly higher for selection differential 1.0 (Table 1, Table S3, Table S4). Similarly, populations with isolated groups at resource levels 50 and 100 and selection differentials 0.1 and 1.0 do not differ significantly in size, but the populations with selection differential 1.0 have a significantly higher number of cultural traits per individual. Populations with large interacting groups under high selection pressure evolve the highest number of cultural traits, as predicted.

**Figure 2:**
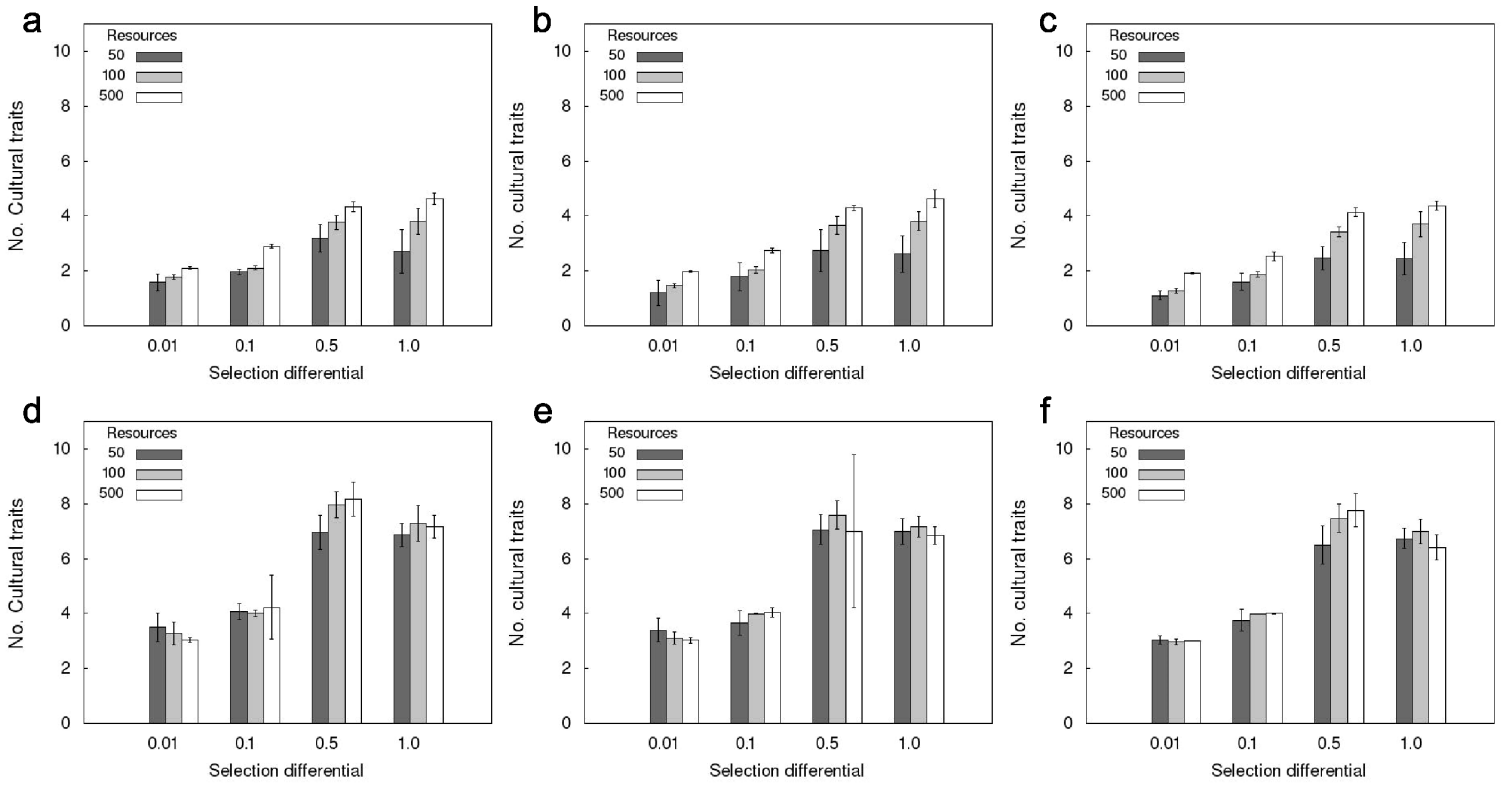
Effect of innovation costs on number of cultural traits per individual. Higher innovation costs can significantly reduce the number of cultural traits per individual, especially in isolated groups with low selection differentials. Maximum energy score of individuals capped at 50. a, d: inventing a new trait costs 10 energy units; b, e: inventing a new trait costs 20 energy units; c, f: inventing a new trait costs: 40 energy units. Upper row: isolated groups, lower row: individuals could learn from or choose partners from neighboring groups; error bars indicate standard deviation; results were compared using Wilcoxon rank sum tests (see Table S7); legend shows max resource value per square.

High innovation costs can significantly reduce the number of cultural traits per individual, especially in populations with isolated groups in which new inventions cannot readily spread (Fig. 2; Table S7, Table S8, Table S9). Learning costs significantly reduced the number of cultural traits in large interacting populations (Fig. 1c, d; Table S10, Table S11, Table S12, Table S13). In populations with isolated groups, the effect of learning costs on the number of cultural traits were only significant for high resource availabilities and if the selection differential was not too high (1.0). In small groups random drift may have been more important than cost-benefit ratios.

Unlike the case where learning was cost-free, populations with interacting groups under very high selection pressure developed a significantly higher number of cultural traits per individual if there was a cost attached to learning. They also had a higher number of cultural traits than populations under lower selection pressure. This may indicate that the additional pressure imposed by learning costs in combination with high benefits (high increase in consumption rate per increase in trait value) compensating for the expenses can drive the evolution of adaptive cultural traits.

We recorded the population growth trajectories for all simulations. For each parameter combination we plotted the mean value of ten runs against time. In many cases we found stable population oscillations or episodes of halted growth superimposed on the overall growth curve (Figure S3). The turning points in each curve correspond to the innovation and spread of a new cultural trait.

## Discussion

In this study, we investigated the relative importance of population size, interaction between groups and natural selection on the rate of evolution of cumulative adaptive culture. We found that high selection pressure can increase cumulative adaptive culture in populations of various sizes and interaction frequencies. Populations of similar size evolved a higher level of cumulative adaptive culture under higher selection pressure. This supports the hypothesis that selection plays a major role in human cultural evolution. If correct, we expect the later Pleistocene expansion of cultural complexity to have been partly driven by selection.

Selection pressure in our model corresponds to population pressure. Keeley [4] defined population pressure as the ratio of population density to the density of available resources. Thus, population pressure can be understood as a form of environmental risk. In a survey of 94 ethnographic hunter-gatherer groups, Keeley found a strong positive correlation between population pressure and socioeconomic complexity, which can be understood as a manifestation of cumulative adaptive culture. Other factors, including population density on its own, did not correlate significantly with socioeconomic complexity, and so he argued that population pressure is a necessary and sufficient condition for socioeconomic complexity.

Two different mechanisms are commonly considered to increase population pressure. One is population growth, and the other a relative decline in environmental resources. It follows that population pressure can occur in poor as well as in rich environments, even though we expect population pressure to be much lower in regions of low resource density compared to high resource density [29]. There is a third way of increasing population pressure: if a sufficient number of individuals within a population increase their efficiency at an adaptive cultural trait, inter-individual competition and population pressure may increase too. This process is called intensification [34], [35], [36], [37]. Our model does not discriminate between these possibilities, but our primary point is that ecological pressure will strongly influence changes in cultural complexity.

In our model increased skill at a cultural trait that leads to a higher per-capita resource extraction rate causes more rapid resource depletion. Populations with isolated groups under higher selection pressure generally had higher levels of competition and more cultural traits than populations under lower selection pressure. In interacting populations competition and trait number declined again if selection differentials were very high. A possible explanation is that cultural group selection is weakened by inter-group transmission. Alternatively, excessive resource pressure may cause a reduction in population size that simultaneously reduces competition and the number of potential innovators. The rate of innovation may also be affected by relative fitness gains [38], [39]. If inventing a new trait leads to relatively smaller fitness gains than improving the efficiency at a pre-existing trait, there is little advantage to innovating independent traits. Consequently, the accumulation of adaptive traits should proceed more slowly. It seems, therefore, that population pressure and population size can interact to increase or decrease the rate of change of cumulative adaptive culture.

The equilibrium population size in a given environment depends in part on the ability of a population to exploit the resources within this environment. Thus, the equilibrium size of a population does not necessarily coincide with the absolute carrying capacity, determined by the sum of all environmental resources, but is usually smaller [29]. Consequently, the distinction between technological improvements that allow individuals to exploit the same resource more efficiently and independent innovations that broaden the subsistence base matters. Only the latter lead to intensive population growth and higher population densities by shifting the equilibrium population size toward the environmental carrying capacity [29], [34], [36]. This is what we see in the population growth trajectories (Figure S3): Innovation of a new trait is followed by exponential population growth that slows down as the new equilibrium population size is reached. This process resembles sequential phases of logistic growth. In populations close to their equilibrium size resource pressure on individuals is high. Consequently, individuals are under pressure to broaden their resource base.

The process of resource intensification in our model closely resembles a “broad spectrum revolution” [38] and has been associated with population pressure and accelerated increases in cultural complexity in various periods of prehistory. Examples include the South African Middle Stone Age [39], the Upper Palaeolithic in Europe and the Near East [40], [41], [42], microlithic innovations in South Asia during the Late Pleistocene [43], and culture change in the Epipalaeolithic leading up to the transition to agriculture [44], [45].

High innovation costs can reduce the level of cumulative adaptive culture. In particular we found that small and isolated populations evolve fewer cultural traits, if the relative fitness gains associated with a new trait are small or the costs are high. If populations are very small and neither the potential fitness gains nor the innovation costs are too high, drift may be a more important driving force of cultural evolution. High relative fitness gains can outweigh high innovation costs. Similarly, increasing the population size or interaction rate can counteract high innovation costs, because of the higher number of potential innovators and the higher rate of cultural diffusion.

The amount of cumulative adaptive culture of a population also depends on the number of previously acquired traits, and the relative costs of maintaining technological traits should have a noticeable effect on cumulative adaptive culture [21], [25]. We can assume that cultural learning, which is necessary for preserving technological skills in a population, is not entirely cost-free, as it takes time and energy. Still, acquiring skills and knowledge by social learning should be more efficient than inventing new skills or technologies by oneself [19], [24], [46].

In our experiments learning costs reduced the level of cumulative adaptive culture of large or connected populations and those under moderately high resource pressure (selection differential 0.5). In small populations drift may have been a more important factor, although learning costs (1 resource unit) were never very high. Surprisingly, populations with very high selection pressure (selection differential 1.0) and interacting groups evolved a significantly higher number of cultural traits if they had to pay a learning cost than if they did not. These results indicate that whether or not adaptive cultural traits will be preserved in a population may depend on relative costs and benefits, as would be expected from behavioural ecology models [47]. As a consequence, a loss of cumulative culture may not necessarily happen by chance or be maladaptive. Rather, expensive traits may disappear from the cultural repertoire of a population, if the costs of preserving them exceed the returns [11], [21], [39], [45].

In many areas of human prehistory the question, what came first, a major cultural innovation or a population expansion, is hitherto unresolved. For example, it is not clear if more complex technological traditions of the African MSA, such as the Still Bay or the Howieson's Poort tradition, were the cause of subsequent population expansions and migrations, or if population growth preceded and perhaps stimulated these cultural innovations [7]. It has also been suggested that both the Still Bay and the Howieson's Poort tradition were reactions to environmental deterioration and ensuing higher population pressure [48], [49], [50], [51].

In our simulations we found a feedback between population dynamics and adaptive cultural evolution that can give rise to population oscillations overlying a general growth trend. Populations expand rapidly following the invention of a new trait, but then come to a halt or even decline during periods of intensified resource exploitation. Population oscillations in our model arise from inherent characteristics of the socio-cultural system and are not induced by external disturbances. Thus, cultural systems may develop their own internal dynamics within their ecological context. In order to explain the complex cultural dynamics of human populations we need to understand the interplay between demographic factors and shifting selective pressures, because they do not occur in isolation from each other. By developing models that explicitly incorporate selection on a behavioural level we suggest we can better integrate the development of the extraordinary human capacity for culture and cultural change with the biological evolution of the species.

## Supporting Information

Figure S1 **Effect of selection pressure, group size and between-group interaction on competition level.** Competition was measured as the fraction of individuals in the total population that cannot meet their resource requirements. Higher selection pressure increases competition up to a point (selection differential 0.5), but beyond this point decreases competition (1.0). The maximum energy score of individuals was capped at 50. a: isolated groups, b: individuals could learn from or choose partners from neighboring groups; results are grouped according to selection differential (x-axis); legend shows max resource value per square.
(TIF)

Figure S2 **Effect of selection pressure, resource availability and between-group interaction on mean group size.** Maximum energy score of individuals capped at 50. Higher resource availability allows for higher group sizes. Higher selection differential allows for faster population growth but limits population size if it is too high. a: isolated groups, b: individuals could learn from or choose partners from neighboring groups; results are grouped according to selection differential (x-axis); legend shows max resource value per square.
(TIF)

Figure S3 **Examples for population oscillations under varying selection pressure and resource availability.** Graphs show mean population sizes of ten independent runs. Turning points in the growth trajectory of populations correspond to the innovation of cultural traits that increase the equilibrium population size shifting it towards the absolute environmental carrying capacity. Max energy score of individuals capped at 50. Neighboring groups were in contact. a) resource level: 50, selection differential (measure for selection pressure) 0.01, innovation cost: 10; b) resource level 500, selection differential 0.1, innovation cost 10; c) resource level 50, selection differential 0.01, innovation cost: 20; d) resource level: 500, selection differential: 0.01, innovation cost: 20; e) resource level: 500, selection differential: 0.1; innovation cost: 20; f) resource level: 100, selection differential: 0.1, innovation cost: 40.
(TIF)

Figure S4 **Process overview and scheduling.** Colour code: pink – processes involving resources, purple – randomisation, blue – individual processes that occur at every time step, green – individual processes that occur conditionally, orange – group process, red – data recording and analysis. E: individual's stored energy, Erep: minimum energy required for reproduction. See text for details. * Only once at model initialisation for time-independent resource distributions.
(TIF)

Table S1 **Results table Wilcoxon-rank-sum test comparison of number of cultural traits per individual for different selection differentials (measure for selection pressure).** Max energy per individual capped at 50. Innovation cost 10 resource units. Bonferroni-correction factor 6 (number of pair-wise tests). Significant results are marked with asterisks. * significant at 0.05; ** significant at 0.01.
(DOCX)

Table S2 **Results table Wilcoxon-rank-sum test comparison of number of cultural traits per individual for different resource availabilities.** Max energy per individual capped at 50. Innovation cost 10 resource units; selection differentials (measure for selection pressure); Bonferroni-correction factor 3 (number of pair-wise tests). Significant results are marked with asterisks. *significant at 0.05; ** significant at 0.01.
(DOCX)

Table S3 **Results table Wilcoxon-rank-sum test comparison of number of cultural traits per individual for populations with isolated or interacting groups at different selection differentials and resource levels.**
(DOCX)

Table S4 **Results table Wilcoxon-rank-sum test comparison of mean group size for different selection differentials (measure for selection pressure).** Max energy per individual capped at 50. Innovation cost 10 resource units. Bonferroni-correction factor 6 (number of tests). Significant results are marked with asterisks. * significant at 0.05; ** significant at 0.01.
(DOCX)

Table S5 **Results table Wilcoxon-rank-sum test comparison of mean group size for different resource availabilities.** Max energy per individual capped at 50. Innovation cost 10 resource units; selection differentials (measure for selection pressure); Bonferroni-correction factor 3 (number of pair-wise tests). Significant results are marked with asterisks. *significant at 0.05; ** significant at 0.01.
(DOCX)

Table S6 **Results table Wilcoxon-rank-sum test comparison of mean group size for populations with isolated or interacting groups at different selection differentials and resource levels.**
(DOCX)

Table S7 **Results table Wilcoxon-rank-sum test comparison of number of cultural traits per individual in populations with max energy per individual capped at 50 and different innovation costs.** Selection differentials (measure for selection pressure). Significant results are marked with asterisks. *significant at 0.05; ** significant at 0.01.
(DOCX)

Table S8 **Mean number of cultural traits and mean group sizes ± standard deviation in populations with isolated or interacting groups, with different selection differentials and resource availabilities.** Maximum energy score of individuals was capped at 50. Cost of inventing a new trait was 20.
(DOCX)

Table S9 **Mean number of cultural traits and mean group sizes ± standard deviation in populations with isolated or interacting groups, with different selection differentials and resource availabilities.** Maximum energy score of individuals was capped at 50. Cost of inventing a new trait was 40.
(DOCX)

Table S10 **Mean number of cultural traits and mean group sizes ± standard deviation in populations with isolated and interacting groups, with different selection differentials and resource availabilities.** Max energy value capped at 50. Cost of inventing a new trait 10 energy units. Learning costs 1 energy unit.
(DOCX)

Table S11 **Results table Wilcoxon-rank-sum test comparison of the number of cultural traits per individual in populations with learning costs (1 resource unit) and without learning costs.** Max energy score per individual capped at 50. Innovation costs 10 resource units. Selection differentials (measure for selection pressure). Significant results are marked with asterisks. *significant at 0.05; ** significant at 0.01.
(DOCX)

Table S12 **Results table Wilcoxon-rank-sum test comparison of number of cultural traits per individual for different selection differentials (measure for selection pressure), if each learning event costs 1 resource unit.** Max energy score per individual capped at 50. Innovation cost 10 resource units. Bonferroni-correction factor 6 (number of pair-wise tests). Significant results are marked with asterisks. * significant at 0.05; ** significant at 0.01.
(DOCX)

Table S13 **Results table Wilcoxon-rank-sum test comparison of mean group size in populations with learning costs (1 resource unit) and without learning costs.** Max energy score per individual capped at 50. Innovation costs 10 resource units. Selection differentials (measure for selection pressure). Significant results are marked with asterisks. *significant at 0.05; ** significant at 0.01.
(DOCX)

Table S14 **Results table Wilcoxon-rank-sum test comparison of total competition for different selection differentials; max energy per individual capped at 50.** Innovation cost 10 resource units. Bonferroni-correction factor 6 (number of pair-wise tests). Significant results are marked with asterisks. * significant at 0.05; ** significant at 0.01.
(DOCX)

Table S15 **Comparison of total competition within a population across different resource levels, keeping selection differential and interaction regime constant.** Max energy per individual capped at 50 energy units. Innovation cost 10 energy units. Pairwise Wilcoxon-rank-sum tests, Bonferroni-correction factor 3 (number of pairwise tests). Significant results are marked with asterisks. * significant at 0.05; ** significant at 0.01.
(DOCX)

Table S16 **Comparison of total competition in a population across different interaction regimes, keeping resource level and selection differential constant.** Max energy per individual capped at 50 energy units. Innovation cost 10 energy units. Pairwise Wilcoxon-rank-sum tests. Significant results are marked with asterisks. * significant at 0.05; ** significant at 0.01.
(DOCX)

Table S17 **Model parameters and used values overview.** * 1 corresponds to within-group searches only, 2 includes groups within the Moore neighbourhood, culture radius and reproductive radius were always varied together.
(DOCX)

Text S1 **ODD (overview, design concepts and details) model description.**
(DOCX)
