# Supplementary figures and images for: High Selection Pressure Promotes Increase in Cumulative Adaptive Culture
Source: PLoS One. 2014 Jan 29;9(1):e86406. doi: 10.1371/journal.pone.0086406 (PMC3906051; doi:10.1371/journal.pone.0086406)

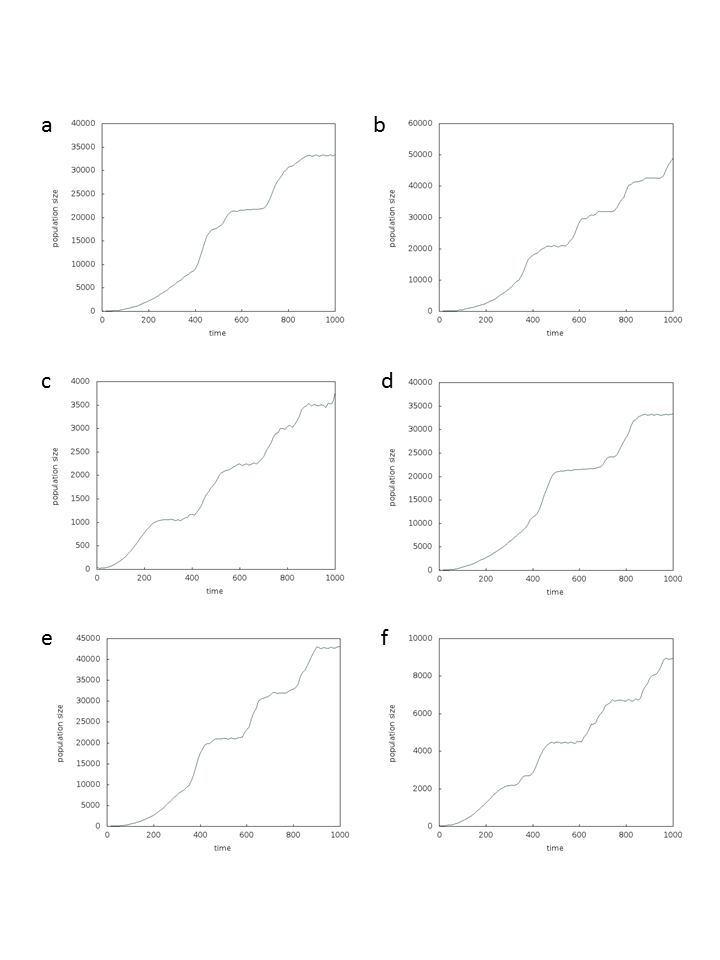

Supplement: Figure S3 — Examples for population oscillations under varying selection pressure and resource availability. Graphs show mean population sizes of ten independent runs. Turning points in the growth trajectory of populations correspond to the innovation of cultural traits that increase the equilibrium population size shifting it towards the absolute environmental carrying capacity. Max energy score of individuals capped at 50. Neighboring groups were in contact. a) resource level: 50, selection differential (measure for selection pressure) 0.01, innovation cost: 10; b) resource level 500, selection differential 0.1, innovation cost 10; c) resource level 50, selection differential 0.01, innovation cost: 20; d) resource level: 500, selection differential: 0.01, innovation cost: 20; e) resource level: 500, selection differential: 0.1; innovation cost: 20; f) resource level: 100, selection differential: 0.1, innovation cost: 40. (TIF) [file pone.0086406.s003.tif]

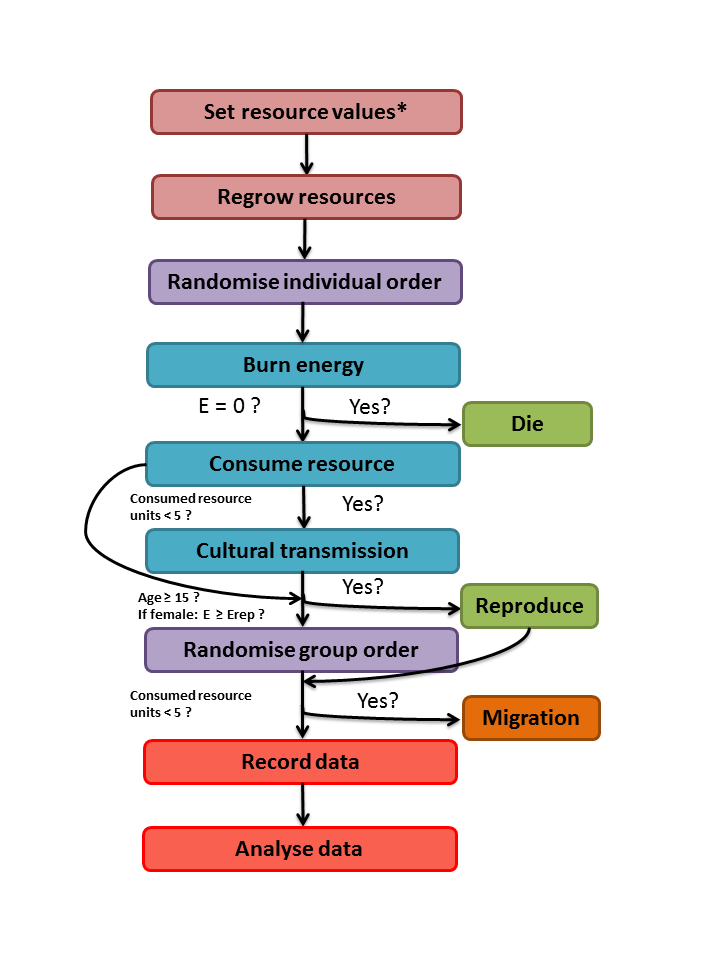

Supplement: Figure S4 — Process overview and scheduling. Colour code: pink – processes involving resources, purple – randomisation, blue – individual processes that occur at every time step, green – individual processes that occur conditionally, orange – group process, red – data recording and analysis. E: individual's stored energy, Erep: minimum energy required for reproduction. See text for details. * Only once at model initialisation for time-independent resource distributions. (TIF) [file pone.0086406.s004.tif]
