# Supplementary material for: High Selection Pressure Promotes Increase in Cumulative Adaptive Culture
Source: PLoS One. 2014 Jan 29;9(1):e86406. doi: 10.1371/journal.pone.0086406 (PMC3906051; doi:10.1371/journal.pone.0086406)
Supplement: Table S1 — Results table Wilcoxon-rank-sum test comparison of number of cultural traits per individual for different selection differentials (measure for selection pressure). Max energy per individual capped at 50. Innovation cost 10 resource units. Bonferroni-correction factor 6 (number of pair-wise tests). Significant results are marked with asterisks. * significant at 0.05; ** significant at 0.01. (DOCX) [file pone.0086406.s005.docx]

| Compare between sel.diffs. | **Isolated groups** | **Interacting groups** |
| --- | --- | --- |
| **Resource level 50** | | |
| 0.01 – 0.1 | 7.578e-05 ** | 0.0007253 ** |
| 0.01 – 0.5 | 1.083e-05 ** | 1.083e-05 ** |
| 0.01 – 1.0 | 1.083e-05 ** | 1.083e-05 ** |
| 0.1 – 0.5 | 1.083e-05 ** | 1.083e-05 ** |
| 0.1 – 1.0 | 1.083e-05 ** | 1.083e-05 ** |
| 0.5 – 1.0 | 0.7394 | 0.0004871 ** |
| **Resource level 100** | | |
| 0.01 – 0.1 | 1.083e-05 ** | 0.0001299 ** |
| 0.01 – 0.5 | 1.083e-05 ** | 1.083e-05 ** |
| 0.01 – 1.0 | 1.083e-05 ** | 1.083e-05 ** |
| 0.1 – 0.5 | 1.083e-05 ** | 1.083e-05 ** |
| 0.1 – 1.0 | 1.083e-05 ** | 1.083e-05 ** |
| 0.5 – 1.0 | 0.02323 | 1.083e-05 ** |
| **Resource level 500** | | |
| 0.01 – 0.1 | 1.083e-05 ** | 1.083e-05 ** |
| 0.01 – 0.5 | 1.083e-05 ** | 1.083e-05 ** |
| 0.01 – 1.0 | 1.083e-05 ** | 1.083e-05 ** |
| 0.1 – 0.5 | 1.083e-05 ** | 1.083e-05 ** |
| 0.1 – 1.0 | 1.083e-05 ** | 0.4813 |
| 0.5 – 1.0 | 0.3527 | 1.083e-05 ** |
