# Supplementary material for: High Selection Pressure Promotes Increase in Cumulative Adaptive Culture
Source: PLoS One. 2014 Jan 29;9(1):e86406. doi: 10.1371/journal.pone.0086406 (PMC3906051; doi:10.1371/journal.pone.0086406)
Supplement: Table S2 — Results table Wilcoxon-rank-sum test comparison of number of cultural traits per individual for different resource availabilities. Max energy per individual capped at 50. Innovation cost 10 resource units; selection differentials (measure for selection pressure); Bonferroni-correction factor 3 (number of pair-wise tests). Significant results are marked with asterisks. *significant at 0.05; ** significant at 0.01. (DOCX) [file pone.0086406.s006.docx]

| **Selection diff.** | **0.01** | **0.1** | **0.5** | **1.0** |
| --- | --- | --- | --- | --- |
| Compare between resource levels | | | | |
| Isolated groups | | | | |
| 50 – 100 | 0.2475 | 0.006841 * | 0.01469 * | 0.00105 ** |
| 50 – 500 | 1.083e-05 ** | 1.083e-05 ** | 1.083e-05 ** | 1.083e-05 ** |
| 100 – 500 | 1.083e-05 ** | 1.083e-05 ** | 4.33e-05 ** | 1.083e-05 ** |
| Interacting groups | | | | |
| 50 – 100 | 0.2729 | 0.6305 | 0.001505 ** | 0.2799 |
| 50 – 500 | 0.1903 | 0.002089 ** | 0.005196 * | 0.7959 |
| 100 – 500 | 0.3845 | 0.0004871 ** | 0.7959 | 0.3642 |
