# Supplementary material for: High Selection Pressure Promotes Increase in Cumulative Adaptive Culture
Source: PLoS One. 2014 Jan 29;9(1):e86406. doi: 10.1371/journal.pone.0086406 (PMC3906051; doi:10.1371/journal.pone.0086406)
Supplement: Table S3 — Results table Wilcoxon-rank-sum test comparison of number of cultural traits per individual for populations with isolated or interacting groups at different selection differentials and resource levels. (DOCX) [file pone.0086406.s007.docx]

| **Selection diff.** | **0.01** | **0.1** | **0.5** | **1.0** |
| --- | --- | --- | --- | --- |
| Resource level | | | | |
| 50 | 1.083e-05 ** | 1.083e-05 ** | 1.083e-05 ** | 1.083e-05 ** |
| 100 | 0.0001817 ** | 0.0001817 ** | 1.083e-05 ** | 1.083e-05 ** |
| 500 | 1.083e-05 ** | 1.083e-05 ** | 1.083e-05 ** | 1.083e-05 ** |
