# Supplementary material for: High Selection Pressure Promotes Increase in Cumulative Adaptive Culture
Source: PLoS One. 2014 Jan 29;9(1):e86406. doi: 10.1371/journal.pone.0086406 (PMC3906051; doi:10.1371/journal.pone.0086406)
Supplement: Table S7 — Results table Wilcoxon-rank-sum test comparison of number of cultural traits per individual in populations with max energy per individual capped at 50 and different innovation costs. Selection differentials (measure for selection pressure). Significant results are marked with asterisks. *significant at 0.05; ** significant at 0.01. (DOCX) [file pone.0086406.s011.docx]

| **Innovation cost** | **10 vs. 20** | | **10 vs. 40** | | **20 vs. 40** | |
| --- | --- | --- | --- | --- | --- | --- |
| **Resource level** | **Isolated groups** | **Interacting groups** | **Isolated groups** | **Interacting groups** | **Isolated groups** | **Interacting groups** |
| Selection differential 0.01 | | | | | | |
| 50 | 0.06301 | 0.393 | 0.001004 ** | 0.0115 * | 0.0113 * | 0.03423 * |
| 100 | 1.083e-05 ** | 0.5706 | 1.083e-05 ** | 0.02569 * | 0.0001299 ** | 0.08921 |
| 500 | 4.33e-05 ** | 0.8534 | 1.083e-05 ** | 0.315 | 2.165e-05 ** | 0.3527 |
| Selection differential 0.1 | | | | | | |
| 50 | 0.7959 | 0.2176 | 0.003886 ** | 0.2799 | 0.005196 ** | 1 |
| 100 | 0.2799 | 0.9705 | 7.578e-05 ** | 0.2475 | 0.008931 ** | 0.3256 |
| 500 | 0.0002057 ** | 0.005196 ** | 2.165e-05 ** | 0.002089 ** | 0.003886 ** | 0.5787 |
| Selection differential 0.5 | | | | | | |
| 50 | 0.1903 | 0.7959 | 0.005196 ** | 0.05243 | 0.1051 | 0.03546 * |
| 100 | 0.6305 | 0.8534 | 0.002089 ** | 0.1051 | 0.06301 | 0.1431 |
| 500 | 0.5787 | 0.7394 | 0.0115 * | 0.5288 | 0.01854 ** | 0.1431 |
| Selection differential 1.0 | | | | | | |
| 50 | 0.9118 | 0.8534 | 0.315 | 0.315 | 0.1903 | 0.07526 |
| 100 | 0.6842 | 0.5787 | 0.393 | 0.2176 | 0.6842 | 0.3642 |
| 500 | 0.6842 | 0.393 | 0.02881 * | 0.005196 ** | 0.05243 | 0.05243 |
