# Supplementary material for: High Selection Pressure Promotes Increase in Cumulative Adaptive Culture
Source: PLoS One. 2014 Jan 29;9(1):e86406. doi: 10.1371/journal.pone.0086406 (PMC3906051; doi:10.1371/journal.pone.0086406)
Supplement: Table S8 — Mean number of cultural traits and mean group sizes ± standard deviation in populations with isolated or interacting groups, with different selection differentials and resource availabilities. Maximum energy score of individuals was capped at 50. Cost of inventing a new trait was 20. (DOCX) [file pone.0086406.s012.docx]

|  | **Isolated groups** | | **Interacting groups** | |
| --- | --- | --- | --- | --- |
| **Resource value** | **Group size** | **No. traits** | **Group size** | **No. traits** |
| Selection differential 0.01 | | | | |
| 50 | 10.22 ± 1.972 | 1.33 ± 0.261 | 37.63 ± 4.733 | 3.40 ± 0.435 |
| 100 | 24.64 ± 1.378 | 1.47 ± 0.071 | 69.79 ± 3.281 | 3.10 ± 0.227 |
| 500 | 183.61 ± 4.425 | 1.99 ± 0.029 | 333.73 ± 8.736 | 3.03 ± 0.105 |
| Selection differential 0.1 | | | | |
| 50 | 17.67 ± 1.700 | 1.96 ± 0.088 | 40.11 ± 4.356 | 3.66 ± 0.437 |
| 100 | 36.97 ± 3.144 | 2.03 ± 0.118 | 89.91 ± 1.477 | 3.99 ± 0.004 |
| 500 | 245.65 ± 10.290 | 2.74 ± 0.087 | 430.60 ± 13.008 | 4.05 ± 0.175 |
| Selection differential 0.5 | | | | |
| 50 | 21.78 ± 2.317 | 2.83 ± 0.264 | 62.48 ± 12.575 | 7.06 ± 0.536 |
| 100 | 56.58 ± 5.342 | 3.65 ± 0.316 | 161.18 ± 12.120 | 7.59 ± 0.512 |
| 500 | 293.87 ± 8.971 | 4.30 ± 0.103 | 808.11 ± 92.771 | 8.25 ± 0.711 |
| Selection differential 1.0 | | | | |
| 50 | 17.38 ± 2.354 | 2.73 ± 0.467 | 41.63 ± 4.823 | 6.98 ± 0.470 |
| 100 | 40.32 ± 2.282 | 3.81 ± 0.339 | 71.01 ± 5.007 | 7.17 ± 0.383 |
| 500 | 225.13 ± 19.455 | 4.63 ± 0.315 | 321.24 ± 20.908 | 6.83 ± 0.327 |
