# Supplementary material for: High Selection Pressure Promotes Increase in Cumulative Adaptive Culture
Source: PLoS One. 2014 Jan 29;9(1):e86406. doi: 10.1371/journal.pone.0086406 (PMC3906051; doi:10.1371/journal.pone.0086406)
Supplement: Table S9 — Mean number of cultural traits and mean group sizes ± standard deviation in populations with isolated or interacting groups, with different selection differentials and resource availabilities. Maximum energy score of individuals was capped at 50. Cost of inventing a new trait was 40. (DOCX) [file pone.0086406.s013.docx]

|  | **Isolated groups** | | **Interacting groups** | |
| --- | --- | --- | --- | --- |
| **Resource value** | **Group size** | **No. traits** | **Group size** | **No. traits** |
| Selection differential 0.01 | | | | |
| 50 | 8.58 ± 1.362 | 1.10 ± 0.169 | 33.19 ± 0.974 | 3.04 ± 0.141 |
| 100 | 21.72 ± 1.014 | 1.28 ± 0.088 | 66.30 ± 4.270 | 2.97 ± 0.097 |
| 500 | 174.41 ± 4.636 | 1.90 ± 0.029 | 330.41 ± 7.092 | 3.00 ± 0.000 |
| Selection differential 0.1 | | | | |
| 50 | 13.17 ± 3.339 | 1.60 ± 0.306 | 41.30 ± 5.339 | 3.76 ± 0.391 |
| 100 | 32.49 ± 2.833 | 1.86 ± 0.109 | 89.31 ± 1.198 | 3.99 ± 0.001 |
| 500 | 227.79 ± 17.360 | 2.53 ± 0.157 | 430.13 ± 12.672 | 4.00 ± 0.022 |
| Selection differential 0.5 | | | | |
| 50 | 19.76 ± 3.211 | 2.46 ± 0.433 | 57.34 ± 13.335 | 6.49 ± 0.692 |
| 100 | 52.68 ± 4.315 | 3.43 ± 0.178 | 158.55 ± 10.557 | 7.47 ± 0.517 |
| 500 | 278.05 ± 11.76 | 4.14 ± 0.171 | 776.50 ± 96.759 | 7.77 ± 0.609 |
| Selection differential 1.0 | | | | |
| 50 | 15.50 ± 3.267 | 2.44 ± 0.595 | 41.50 ± 4.360 | 6.74 ± 0.357 |
| 100 | 40.21 ± 4.378 | 3.71 ± 0.463 | 71.37 ± 6.133 | 6.98 ± 0.450 |
| 500 | 211.89 ± 7.47 | 4.38 ± 0.166 | 304.78 ± 33.322 | 6.41 ± 0.464 |
