# Supplementary material for: High Selection Pressure Promotes Increase in Cumulative Adaptive Culture
Source: PLoS One. 2014 Jan 29;9(1):e86406. doi: 10.1371/journal.pone.0086406 (PMC3906051; doi:10.1371/journal.pone.0086406)
Supplement: Table S10 — Mean number of cultural traits and mean group sizes ± standard deviation in populations with isolated and interacting groups, with different selection differentials and resource availabilities. Max energy value capped at 50. Cost of inventing a new trait 10 energy units. Learning costs 1 energy unit. (DOCX) [file pone.0086406.s014.docx]

|  | **Isolated groups** | | **Interacting groups** | |
| --- | --- | --- | --- | --- |
| **Resource value** | **Group size** | **No. traits** | **Group size** | **No. traits** |
| Selection differential 0.01 | | | | |
| 50 | 10.12 ± 1.639 | 1.40 ± 0.229 | 33.09 ± 3.678 | 3.17 ± 0.368 |
| 100 | 26.65 ± 2.044 | 1.65 ± 0.107 | 65.10 ± 0.692 | 3.00 ± 0.001 |
| 500 | 183.86 ± 6.254 | 2.04 ± 0.035 | 321.1 ± 2.952 | 3.00 ± 0.001 |
| Selection differential 0.1 | | | | |
| 50 | 14.80 ± 3.408 | 1.79 ± 0.328 | 46.78 ± 3.878 | 3.75 ± 0.343 |
| 100 | 31.13 ± 2.722 | 1.98 ± 0.074 | 76.39 ± 7.849 | 3.78 ± 0.414 |
| 500 | 232.79 ± 7.907 | 2.71 ± 0.092 | 381.36 ± 45.166 | 3.80 ± 0.400 |
| Selection differential 0.5 | | | | |
| 50 | 19.49 ± 2.479 | 2.65 ± 0.336 | 36.90 ± 3.689 | 5.90 ± 0.568 |
| 100 | 48.78 ± 3.538 | 3.21 ± 0.207 | 104.84 ± 7.390 | 6.36 ± 0.494 |
| 500 | 288.21 ± 14.054 | 4.07 ± 0.105 | 523.27 ± 32.643 | 6.73 ± 0.413 |
| Selection differential 1.0 | | | | |
| 50 | 15.87 ± 1.763 | 2.60 ± 0.367 | 48.28 ± 2.535 | 7.17 ± 0.403 |
| 100 | 41.10 ± 4.329 | 3.71 ± 0.311 | 106.96 ± 5.689 | 8.58 ± 0.466 |
| 500 | 222.10 ± 12.317 | 4.51 ± 0.198 | 533.46 ± 40.457 | 8.78 ± 0.578 |
