# Supplementary material for: High Selection Pressure Promotes Increase in Cumulative Adaptive Culture
Source: PLoS One. 2014 Jan 29;9(1):e86406. doi: 10.1371/journal.pone.0086406 (PMC3906051; doi:10.1371/journal.pone.0086406)
Supplement: Table S11 — Results table Wilcoxon-rank-sum test comparison of the number of cultural traits per individual in populations with learning costs (1 resource unit) and without learning costs. Max energy score per individual capped at 50. Innovation costs 10 resource units. Selection differentials (measure for selection pressure). Significant results are marked with asterisks. *significant at 0.05; ** significant at 0.01. (DOCX) [file pone.0086406.s015.docx]

| **Resource value** | **Isolated groups** | **Interacting groups** |
| --- | --- | --- |
| **Selection differential 0.01** | | |
| **50** | 0.1051 | 0.03546 * |
| **100** | 0.0115 * | 0.001004 ** |
| **500** | 0.01469 * | 0.1051 |
| **Selection differential 0.1** | | |
| **50** | 0.2799 | 0.01553 * |
| **100** | 0.003886 ** | 0.1051 |
| **500** | 4.33e-05 ** | 0.0003281 ** |
| **Selection differential 0.5** | | |
| **50** | 0.008931 ** | 0.0002057 ** |
| **100** | 0.0001299 ** | 1.083e-05 ** |
| **500** | 0.0007253 ** | 0.001004 ** |
| **Selection differential 1.0** | | |
| **50** | 0.5288 | 0.3445 |
| **100** | 0.4359 | 0.0007253 ** |
| **500** | 0.1903 | 4.33e-05 ** |
