# Supplementary material for: High Selection Pressure Promotes Increase in Cumulative Adaptive Culture
Source: PLoS One. 2014 Jan 29;9(1):e86406. doi: 10.1371/journal.pone.0086406 (PMC3906051; doi:10.1371/journal.pone.0086406)
Supplement: Table S12 — Results table Wilcoxon-rank-sum test comparison of number of cultural traits per individual for different selection differentials (measure for selection pressure), if each learning event costs 1 resource unit. Max energy score per individual capped at 50. Innovation cost 10 resource units. Bonferroni-correction factor 6 (number of pair-wise tests). Significant results are marked with asterisks. * significant at 0.05; ** significant at 0.01. (DOCX) [file pone.0086406.s016.docx]

| Compare between sel.diffs. | **Isolated groups** | **Interacting groups** |
| --- | --- | --- |
| **Resource level 50** | | |
| 0.01 – 0.1 | 0.005196 * | 0.002879 ** |
| 0.01 – 0.5 | 1.083e-05 ** | 1.083e-05 ** |
| 0.01 – 1.0 | 1.083e-05 ** | 0.0001817 ** |
| 0.1 – 0.5 | 2.165e-05 ** | 1.083e-05 ** |
| 0.1 – 1.0 | 4.33e-05 ** | 0.0001817 ** |
| 0.5 – 1.0 | 1 | 0.0003281 ** |
| **Resource level 100** | | |
| 0.01 – 0.1 | 2.165e-05 ** | 0.002089 * |
| 0.01 – 0.5 | 1.083e-05 ** | 1.083e-05 ** |
| 0.01 – 1.0 | 1.083e-05 ** | 1.083e-05** |
| 0.1 – 0.5 | 1.083e-05 ** | 1.083e-05 ** |
| 0.1 – 1.0 | 1.083e-05 ** | 1.083e-05 ** |
| 0.5 – 1.0 | 0.001505 ** | 1.083e-05 ** |
| **Resource level 500** | | |
| 0.01 – 0.1 | 1.083e-05 ** | 0.0001817 ** |
| 0.01 – 0.5 | 1.083e-05 ** | 0.0001817 ** |
| 0.01 – 1.0 | 1.083e-05 ** | 1.083e-05 ** |
| 0.1 – 0.5 | 1.083e-05 ** | 0.0001806 ** |
| 0.1 – 1.0 | 1.083e-05 ** | 0.0001817 ** |
| 0.5 – 1.0 | 4.33e-05 ** | 0.0001817 ** |
