# Supplementary material for: High Selection Pressure Promotes Increase in Cumulative Adaptive Culture
Source: PLoS One. 2014 Jan 29;9(1):e86406. doi: 10.1371/journal.pone.0086406 (PMC3906051; doi:10.1371/journal.pone.0086406)
Supplement: Table S15 — Comparison of total competition within a population across different resource levels, keeping selection differential and interaction regime constant. Max energy per individual capped at 50 energy units. Innovation cost 10 energy units. Pairwise Wilcoxon-rank-sum tests, Bonferroni-correction factor 3 (number of pairwise tests). Significant results are marked with asterisks. * significant at 0.05; ** significant at 0.01. (DOCX) [file pone.0086406.s019.docx]

| **Selection diff.** | **0.01** | **0.1** | **0.5** | **1.0** |
| --- | --- | --- | --- | --- |
| Compare between resource levels | | | | |
| Isolated groups | | | | |
| 50 – 100 | 0.2176 | 0.5787 | 0.0004871 ** | 0.2475 |
| 50 – 500 | 1.083e-05 ** | 0.03546 | 1.083e-05 ** | 1.083e-05 ** |
| 100 – 500 | 0.0007253 ** | 0.01469 * | 1.083e-05 ** | 1.083e-05 ** |
| Interacting groups | | | | |
| 50 – 100 | 1.083e-05 ** | 0.00105 ** | 0.1655 | 0.0001299 ** |
| 50 – 500 | 1.083e-05 ** | 0.0007253 ** | 0.9705 | 1.083e-05 ** |
| 100 – 500 | 0.00105 ** | 0.315 | 0.02323 | 1.083e-05 ** |
