# Supplementary material for: High Selection Pressure Promotes Increase in Cumulative Adaptive Culture
Source: PLoS One. 2014 Jan 29;9(1):e86406. doi: 10.1371/journal.pone.0086406 (PMC3906051; doi:10.1371/journal.pone.0086406)
Supplement: Table S16 — Comparison of total competition in a population across different interaction regimes, keeping resource level and selection differential constant. Max energy per individual capped at 50 energy units. Innovation cost 10 energy units. Pairwise Wilcoxon-rank-sum tests. Significant results are marked with asterisks. * significant at 0.05; ** significant at 0.01. (DOCX) [file pone.0086406.s020.docx]

| **Selection diff.** | **0.01** | **0.1** | **0.5** | **1.0** |
| --- | --- | --- | --- | --- |
| Resource level | | | | |
| 50 | 1.083e-05 ** | 4.33e-05 ** | 2.165e-05 ** | 0.1431 |
| 100 | 0.0003248 ** | 0.0002057 ** | 1.083e-05 ** | 0.3527 |
| 500 | 1.083e-05 ** | 1.083e-05 ** | 1.083e-05 ** | 0.08921 |
