# Supplementary material for: High Selection Pressure Promotes Increase in Cumulative Adaptive Culture
Source: PLoS One. 2014 Jan 29;9(1):e86406. doi: 10.1371/journal.pone.0086406 (PMC3906051; doi:10.1371/journal.pone.0086406)
Supplement: Table S17 — Model parameters and used values overview. * 1 corresponds to within-group searches only, 2 includes groups within the Moore neighbourhood, culture radius and reproductive radius were always varied together. (DOCX) [file pone.0086406.s021.docx]

| Parameters | Values |
| --- | --- |
| Number of Cells X x Y | 10 x 10 |
| Initial population size | 50 |
| Maximum value of a resource | 50, 100, 500 |
| Numbers of resources per cell | 10 |
| Initial energy value of a human agent | 15 |
| Maximum energy value of an individual | 50 |
| Metabolic rate | 4 |
| Basic resource consumption rate | 5 |
| Energy value required for reproduction | 6 |
| Energy passed on to offspring | 5 |
| Maximum number of cultural traits | 10 |
| Maximum skill level at a cultural trait | 10 |
| Cultural transmission error | 5 |
| Migration radius | 2 * |
| Culture radius | 1, 2 * |
| Reproductive radius | 1, 2 * |
| Minimum age for reproduction | 15 |
| Maximum age | 50 |
| Selection differential | 0.01, 0.1, 0.5, 1.0 |
| Innovation costs | 10, 20, 40 |
| Learning costs | 0, 1 |
| Number of time steps | 1000 |
| Number of iterations | 10 |
