# Supplementary material for: High Selection Pressure Promotes Increase in Cumulative Adaptive Culture
Source: PLoS One. 2014 Jan 29;9(1):e86406. doi: 10.1371/journal.pone.0086406 (PMC3906051; doi:10.1371/journal.pone.0086406)
Supplement: Text S1 — ODD (overview, design concepts and details) model description. (DOCX) [file pone.0086406.s022.docx]

**Text S1: Model Outline**

*Models are described according to the ODD standard protocol for agent-based models suggested by* [1].

**Overview**

The purpose of this model is to assess how population size, interaction between different human groups and intensity of selection pressure affect the level of cumulative adaptive culture in human populations. We measure the level of cumulative adaptive culture of a population as the mean number of cultural traits per individual.

State variables and scales: The virtual environment consists of a two-dimensional toroidal landscape (to avoid edge effects) which is partitioned into 10 x 10 individual squares. Each square contains the same amount of ten different resources. Information about resources is stored in vectors that belong to each square.

Human groups are modelled as agents that contain a variable number of individuals. At each time step one group occupies one square in the landscape. Each human individual consumes a specified amount of environmental resources per time step. If the size of a group exceeds the carrying capacity of the square, the groups can fission and/or migrate over a distance defined by the migration radius.

Humans are represented by individual agents that can be either male or female and live up to 50 time steps. Every human agent metabolises a fixed amount of resource energy per time step. Surplus energy is stored and added to an individual’s energy score. The maximum energy score of an individual was capped at 50 energy units. Each individual at first has the same basic proficiency at the same cultural trait that enables him/her to exploit the same specified environmental resource at the basic resource consumption rate. Through innovation or social learning, individuals can acquire up to ten cultural traist - one for each resource – that are stored in the cultural trait vector of an individual [2]. Cultural traits correspond to a specified position in the cultural trait vector. The variants of each cultural trait are represented by integer values in the range of one to ten. The numerical value of each variant can be directly converted into a specific level of efficiency at extracting a particular resource by the following equation:

$$consumption rate=basic rate+\left( trait value-1 \right) \times selection differential$$

The consumption rate corresponds to the resource extraction efficiency. The basic rate is the resource-specific consumption rate if the cultural trait variant has a value of one (minimum resource requirement). The selection differential is a key model parameter that determines the level of competition for resources and thereby the intensity of selection. Individuals that have a higher numerical value at a particular trait variant consume more units of the specified resource per time step. The copying error rate in the transmission of cultural traits is five percent. This means that with probability five percent a learner acquires a cultural trait that is one unit larger or smaller than the same trait in the cultural model (step-wise cultural change).

Process overview and scheduling: At each time step every agent passes through the following processes in the given order: 1) burning energy, 2) resource consumption, 3) learning if the agent is under resource stress, 4) reproduction if the agent’s age is at least fifteen time steps and the agent has a minimum stored energy value of six resource units, and 5) migration (see migration submodel). The computer can only process agents sequentially. But to simulate parallel processing the order in which individual agents are processed is randomised at the beginning of each computational time step. The order in which group agents are processed is randomised before the migration submodel starts.

Agents burn five energy units per time step and consume resources according to their current resource consumption rate. At each time step, each individual only consumes one resource at the rate specified by the equation above. If one resource is not enough to meet the minimum resource requirement, individuals attempt to consume resources in the order that they are assigned in the resource vector and at the individual’s specified consumption rate for that resource. If an individual does not have a trait that enables it to consume a specified resource, it cannot consume this resource. If no attainable resources are available, the individual does not consume any resources at that time step.

If an agent runs out of energy, it dies and is removed from the simulation. Agents are also removed from the simulation when they reach their maximum age of fifty time steps. Resource energy is converted one-to-one into metabolic energy. Any unburnt resource energy is added to the agent’s energy store.

From the age of fifteen agents can choose a partner from within their own group and/or from other groups, depending on the parameter settings. Partners are chosen at random and do not stay together in the following time step. Mature female agents can give birth to one offspring per time step. Each offspring receives five resource units from the mother’s energy store at birth. Offspring inherit all cultural traits present in either of their parents. If a trait is present in both parents, but one parent has a higher-value variant, offspring inherit the higher-value variant. The copy error in cultural transmission from parent to offspring is five percent.

If an individual does not meet his/her minimum resource requirements, he/she randomly chooses another individual as a model (either within his/her own group or within the Moore neighbourhood of the group) and copies the cultural trait of the model with a copy error of five percent. This means that individuals only invest in the acquisition of new or improved cultural skills if they are under resource stress and that there is no bias for improvement during imperfect copying.

Once an individual has reached the highest possible value that a variant of a cultural trait can reach in our simulation, he/she is able to invent a new cultural trait that will enable him/her to exploit a new resource. Thus, we assume that a certain level of technical experience is necessary to invent a new trait. The invention of a new trait comes at a cost that is subtracted from the individual’s energy score. If individuals do not have a high enough amount of stored energy, they cannot invent a new trait, because innovation is too expensive. Immediately after invention, a new trait is always present as a low-efficiency variant that allows a carrier to consume the new resource at the basic rate. Via learning and imperfect copying more efficient variants of the trait can be invented. Equally, a trait can be lost again, if it is reset to zero.

There can only be one group of agents per square. The group can become as large as the carrying capacity of the square (its summed resource values) allows. Agents from within the same group that do not meet their resource requirements can form a new group and migrate to another square. Migration follows the rules detailed below in the migration submodel.

The simulation runs for 1000 time steps. Every 10 time steps, information is collected on the number of occupants in each square (group size) and the cultural traits of each agent in each occupied square. From these values the following characteristics are calculated: total population size, competition level, and the number of cultural traits per individual. The level of competition within the population is measured as the fraction of individuals that cannot meet their minimum resource requirements at a given time step.

In order to account for stochastic variation, we included ten independent simulation runs and used different random number seeds for each run. We only included runs where populations did not go extinct early due to random effects (for example randomly skewed male-female ratio). In the bar charts we plot the mean of the end values of ten independent simulation runs and the standard deviation.

**Design concepts**

Cumulative adaptive culture: Cumulative adaptive culture arises through the accumulation of skills and knowledge over time via cultural transmission from one generation to the next. In our simulation model, individuals learn cultural traits from their parents and from other individuals within their social group. Consequently, individuals do not have to invent all traits anew every generation. Through incorrect copying, individuals can change their proficiency at a cultural skill, so it can be either one unit higher or lower. If individuals have sufficient energy reserves and experience at some cultural skill, they can also invent a new cultural trait that others can acquire by social learning.

Subsistence intensification/extensification: Our model focuses on subsistence-related cultural traits as their adaptive value is relatively easy to assess. The innovation of cultural traits that allow individuals to exploit a new resource increases the carrying-capacity of a habitat and can be regarded as a form of intensification. In contrast, the improvement of existing cultural traits that allow individuals to exploit bigger amounts of a known resource does not increase the carrying capacity of a habitat and can even decrease it. The integration of different resources into the subsistence spectrum culminates in a process known as “broad spectrum revolution” [3] that can be the result of increased population pressure and competition. Cultural changes associated with a broad spectrum revolution or resource intensification may therefore be driven by competition.

Population pressure: has been defined as the ratio of population density to resource density for a given population in a given area and has been shown to correlate with various indicators for cultural complexity in an ethnographic sample [4]. In our model population pressure arises because of two factors: population growth and the increased capacity of individuals to extract more resources due to technological improvements. The selection differential is a model parameter that determines how much more of a resource an individual can extract after improving its subsistence technology by one unit. Greater selection differentials lead to faster resource depletion, increased inter-individual competition and greater population pressure.

Sensing: Individual agents and group agents have a radius of perception within which they search for new resource patches. Individual agents moreover have a radius within which they can search for cultural models and a radius within which they can search for mating partners. Agents “perceive” the age, sex and energy state of potential reproductive partners, the cultural traits of other agents and the resource value of surrounding squares.

Stochasticity: There are several sources of stochasticity in the model operating at two levels. On the individual level, partners for cultural exchange and reproduction are encountered randomly. On the trait level, transmission of cultural traits is incorrect with probability five percent.

Emergence: The autonomous and uncoordinated behaviour of individual agents gives rise to population-level phenomena, such as the number and distribution of cultural traits. The average number of cultural traits per individual is affected by the interaction of individual agents, the amount of resources in the environment and selection pressure.

Data collection: Data is collected at the group level (number of occupied squares, equivalent to the number of human groups), the individual level (number of agents in each square) and the cultural trait level (value of cultural variant for each trait of each agent) to calculate population-level characteristics (mean number of cultural traits per individual, mean cumulative culture index per individual, competition level).

**Details**

Initialisation: Initial resource values are the same for each square. The simulation starts with one group of fifty agents. At the beginning, all agents have one cultural trait that enables them to exploit one specified resource at the basic consumption rate. Agents of the first generation start out as adults with energy reserves of fifteen resource units. Sex is allocated randomly with an equal probability of fifty percent as male or female.

Submodels:

Resource growth: Resources that have been consumed in the previous time step are re-grown at the beginning of the subsequent time step, so that at the beginning of each time step the amount of resources is constant.

Migration: If the resource abundance of a square is too low to support all of its inhabitants, those individuals within the group that could not meet their resource requirements migrate to the most resource-rich, unoccupied square within their radius of perception.

If there is no unoccupied square within the radius of perception, but there are one or more squares occupied by fewer agents than there are members in the migrating group, the migrating group chooses the square occupied by the smallest group and replaces the smaller group. The same rule applies if the only unoccupied squares within the radius of perception have a resource value of zero. If a group about to migrate finds itself in a situation where it can move to several squares of equal quality, one of the available squares is chosen at random. If no square is available to the potential migrants, they have to stay with their original group. Agents can survive for a time living of their stored energy without taking in resource energy. However, once their energy reserves are used up, agents die and are removed from the simulation.

**References**

1. Grimm V, Berger U, Bastiansen F, Eliassen S, Ginot V et al. (2006) A standard protocol for describing individual-based and agent-based models. Ecol. Model. 198: 115-126.
2. Premo LS, Kuhn SL (2010) Modeling effects of local extinctions on culture change and diversity in the Paleolithic. PLOS ONE 5: e15582.
3. Flannery KV (1969) Origins and ecological effects of early domestication in Iran and the Near East. In: The domestication and exploitation of plants and animals. Ucko PJ Dimbelby GW, editors. Chicago: Aldine. pp. 73-100.
4. Keeley LH (1988) Hunter-gatherer economic complexity and “population pressure”: a cross-cultural analysis. J. Anthropol. Archaeol. 7: 373-411.
